# MS/MS library facilitated MRM quantification of native peptides prepared by denaturing ultrafiltration

**DOI:** 10.1186/1477-5956-10-7

**Published:** 2012-02-04

**Authors:** Juraj Lenco, Renny Lan, Nathan Edwards, Radoslav Goldman

**Affiliations:** 1 Georgetown University, Department of Oncology, Lombardi Comprehensive Cancer Center, 3970 Reservoir Rd NW, Washington, DC 20057, USA; 2 Georgetown University, Department of Biochemistry and Molecular and Cellular Biology, Washington, DC, USA; 3 Current address: University Hospital Hradec Kralove; Center for Biomedical Research; Sokolska 581; 500 05 Hradec Kralove; Czech Republic

**Keywords:** Native Peptides, denaturing ultrafiltration, MS/MS library, MRM quantitation

## Abstract

Naturally occurring native peptides provide important information about physiological states of an organism and its changes in disease conditions but protocols and methods for assessing their abundance are not well-developed. In this paper, we describe a simple procedure for the quantification of non-tryptic peptides in body fluids. The workflow includes an enrichment step followed by two-dimensional fractionation of native peptides and MS/MS data management facilitating the design and validation of LC- MRM MS assays. The added value of the workflow is demonstrated in the development of a triplex LC-MRM MS assay used for quantification of peptides potentially associated with the progression of liver disease to hepatocellular carcinoma.

## Background

Proteolysis is an important but perhaps the most overlooked eukaryotic post-translational modification. The biology of neuropeptides [1], peptide hormones [2,3], and unusual proteolytically derived signaling molecules [4,5] stimulates interest in the establishment of appropriate analytical workflows. Mass spectrometry is one of the most useful methods for the analysis of complex peptide mixtures. Proteomic assays typically utilize sequence-specific proteases to characterize the components of complex protein mixtures [6] but the methods for analysis of naturally occurring peptides, without a proteolytic step, are less developed. Applications of mass spectrometry to the study of peptides in various body fluids including cerebrospinal fluid [7], urine [8], synovial fluid [9], saliva [10] and of course serum and plasma [11,12] have been described. A universally useful method for the preparation of the peptides for analysis has not yet emerged and context-specific optimization is typically required. The reported methods include ultrafiltration [13], precipitation by organic solvents [14], solid phase extraction [15], size-exclusion chromatography [11], differential solubilization method [16], and nanoparticle trapping technology [17]. Even methods as simple as direct MALDI-TOF analysis of a complex mixture in a body fluids were used successfully [12] with the benefit of high-throughput, minimal preparative losses of analytes, and minimal sample requirements. Indeed, MALDI- or SELDI-TOF based analyses are most likely the richest source of information about native peptides. On the other hand, these methods suffer from inherent quantitative limitations [15,18]. The original semi quantitative screens are therefore followed by the development of isotope dilution kinetic assays [19] and, most recently, multiple reaction monitoring (MRM) LC-MS/MS quantification of target peptides [20].

MRM has emerged as an LC-MS alternative to antibody based assays for accurate protein quantification [21]. This targeted technology for monitoring of select proteins in complex matrices exploits the sensitivity and selectivity of triple quadrupole mass spectrometers. Specific combinations of precursor *m/z *and its fragments (called transitions) are monitored with linear quantification across several orders of magnitude. Perhaps the most valuable feature of the assays is the ability to multiplex hundreds of analytes [22]. The design and validation of new MRM assays, however, is labor and cost intensive if information about the fragmentation of the peptides of interest is not available. For protein quantification using a tryptic peptide, empirical data stored in PeptideAtlas may be exploited [23]. PeptideAtlas lists peptides most frequently observed in proteomic studies and their most abundant fragments. Unfortunately, such an information-rich database is not available for native peptides and minimal data applicable to ESI ionization are publically available. This substantially increases the number of potential transitions that must be considered in assay design, to account for multiple precursor charge states, the high number of potential fragments, and their different charge states.

Our overall goal was to develop a workflow leading from standardized preparation of serum samples, through data management facilitating the design of MRM quantification, to the validation in a clinically applicable assay. We have therefore developed and characterized a simple method that efficiently inhibits proteolytic processes in body fluids, with sufficient peptide recovery for LC-MRM MS quantification. To facilitate the design of the MRM assays, we established a Skyline [24] library of MS/MS spectra of native serum peptides. To our best knowledge, this is the first attempt to use 2D-HPLC ESI-MS/MS to build a MS/MS library of native peptides in order to facilitate the design of quantitative MRM assays. As a proof-of-concept, the library was applied to the MRM assay development for three peptides with diagnostic potential in liver cirrhosis and hepatocellular cancer [25-27]. This workflow is not specific to hepatocellular cancer, and is expected to facilitate the development of quantitative LC-MS-MRM assays of native peptides in other studies as well. Specific experimental endpoints may require different preparative steps but we believe that the establishment of a publically available library of MS/MS spectra will facilitate rapid screening of target analytes in clinically relevant samples. This is expected to improve the ability of researchers to validate previously suggested or newly discovered biomarker candidates.

## Results and Discussion

Quantification of peptides in biological samples is of general interest [2,5,11]. The mass spectrometric MRM quantification of peptides has become a viable alternative to the traditional ELISA assays [28,29]. MRM quantification is an appealing alternative particularly for multiplex assays of proteolytically modified peptides, such as the native peptides discussed in this article. A discussion of the biology of the native peptides is beyond the scope of this paper; we do not make any claims as to physiological or diagnostic relevance of the peptides analyzed here. We focus on the presentation of a general method for peptide extraction from body fluids and on the generation of native peptide MS/MS library to facilitate the design of quantitative assays for the exploration of the biology of native peptides. It is hard to imagine that a specific quantitative ELISA assay for each of the peptides can be developed; LC-MRM MS is an inviting alternative in this context [28]. We have therefore first optimized a denaturing ultrafiltration method for stabilization and extraction of the native peptides in body fluids and then used the protocol to create a library of MS/MS spectra that helped us optimize efficiently the MRM quantification of three native peptides.

### Effect of different solvents on peptide yield

**Figure 1:**
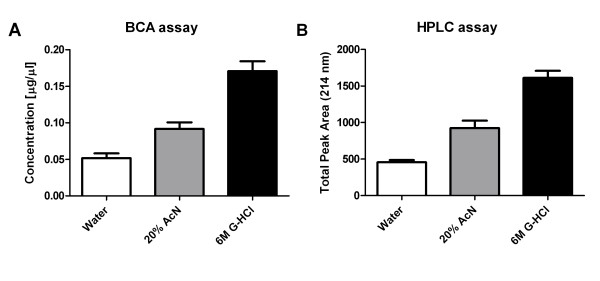
**BCA (A) and HPLC-UV (B) quantification of peptides in the ultrafiltrates prepared in water, 20% Acetonitrile, or 6 M guanidine HCl**. Each sample was prepared in triplicate.

Achieving stability of peptides in body fluids is a common quantitative challenge [28,30]. We have shown by two independent methods that the yield of peptides from serum stabilized with 6 M guanidine hydrochloride (guanidine HCl; G-HCl in figures) is higher than the yield from serum stabilized with acetonitrile (AcN) (Figure 1). Dilution with water led to about 3× lower yield compared to guanidine-HCl while AcN at 20% partially improved recovery. This is likely related to proteolytic degradation of the peptides and (non)specific adsorption of native peptides on surfaces and carrier proteins. The highly denaturing guanidine HCl conditions inactivate trypsin and other proteases and disrupt nonspecific binding of peptides to carrier proteins such as albumin [31] and high-density lipoprotein [32]. Samples containing guanidine HCl can be efficiently desalted by C18 solid phase extraction or directly during well-designed reverse phase chromatography. The results suggest that stabilization of the peptides with 6 M guanidine HCl is more efficient than 20% AcN.

**Figure 2:**
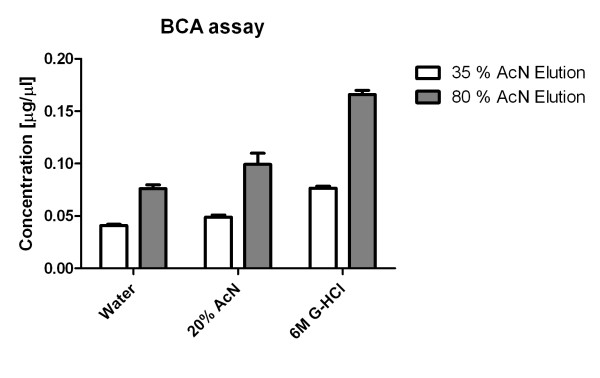
**Polypeptide concentration in the ultrafiltrates eluted with 35% and 80% AcN following C18-SPE desalting**. Each sample was prepared in triplicate.

The ultrafiltration membranes are an efficient enrichment device in combination with the guanidine HCl denaturation, but it must be pointed out that the cutoff values are not precise; loss of some peptides below the cutoff and leakage of proteins with mass above the cutoff seems unavoidable. The membranes were designed to retain polypeptides above the cutoff; but the recovery of peptides from the filtrates must be verified and optimized. We find that both saturation of non-specific binding sites with carrier proteins such as bovine serum albumin (BSA) and the guanidine HCl denaturing conditions improve recovery. Other studies have shown that the filtrate may be contaminated by proteins above the mass cut-off of the filters [33]. We have therefore fractionated the ultrafiltrates by HPLC, using a ProSwift RP-1S 4.6 × 50 mm monolithic column. Native peptides were detected using MALDI-TOF MS only in early fractions. A standard in-solution tryptic digestion of the later eluting fractions followed by LC-MS/MS identified tryptic peptides of albumin, alpha-2-HS glycoprotein, apolipoprotein A-I and apopolipoprotein C-III. These contaminants eluted from the reverse phase monolith at higher AcN concentrations. Consequently, we used 35% instead of initial 80% AcN for elution of peptides from the RP SPE cartridges to further enrich low molecular weight peptides. The chromatograms of ultrafiltrates eluted with 35% AcN show the removal of the large polypeptide fraction without affecting the recovery of low molecular weight peptides. SDS Bis-Tris gel electrophoresis of proteins further confirmed that 35% organic SPE elution solvent helped with removal of residual proteins whose bands were detected in the ultrafiltrates eluted from SPE in 80% AcN. This was accompanied by a decrease of the ultrafiltrate polypeptide concentration measured by BCA assay (Figure 2). The large polypeptides at this low concentration do not directly interfere with the LC-MS analysis of native peptides but can lead to overestimates of the peptide concentration.

### Test of denaturing conditions in a BSA/trypsin model and in serum

**Figure 3:**
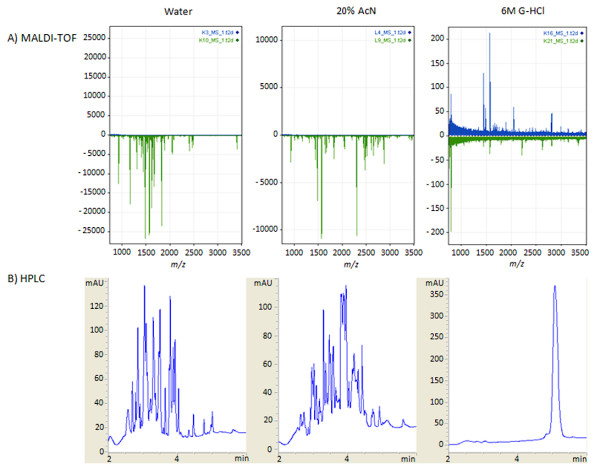
**MALDI-TOF (A) and HPLC-UV (B) analysis of tryptic digests of BSA in water, 20% AcN, and 6 M guanidine HCl**. The inverted spectra in panel A represent digests with trypsin compared to an appropriate solvent control. HPLC-UV chromatograms in panel B show peptide peaks after trypsin digestion in water and 20% AcN and the parent peak for undigested BSA protein in the presence of 6 M guanidine HCl. Each sample was prepared in triplicate and one representative spectrum or chromatogram is presented

To evaluate the inhibition of proteolysis by the guanidine HCl, we used a BSA trypsin digest model. MALDI-TOF MS analysis demonstrates that guanidine HCl at a 6 mol/l concentration stops the catalytic activity of trypsin (Figure 3). The denaturing activity of 20% AcN, used for this purpose in other studies, including ours [13,14], is significantly lower, as trypsin clearly retains activity under these conditions. The concentration of AcN cannot be increased in this setting because higher concentrations are not compatible with the ultrafiltration membrane. The MALDI-TOF results were confirmed by HPLC-UV chromatograms; again, the tryptic activity is similar in the water solvent control and with the addition of 20% AcN (Figure 3b). Proteolysis is inhibited with 6 M guanidine HCl, where the parent protein remains intact.

**Figure 4:**
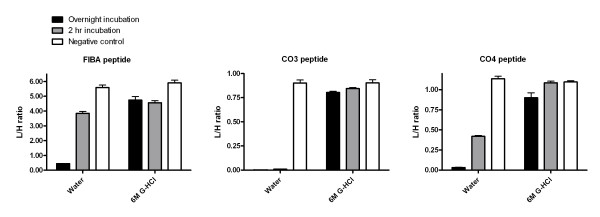
**Recovery of three peptides (FIBA, CO3. and CO4) from serum following 2 h and overnight incubations with the addition of 6 M guanidine HCl or water**. Each sample was prepared in triplicate.

To confirm the denaturing efficiency of 6 M guanidine HCl in serum, we have used heavy analogs of FIBA, CO3, CO4 (fibrinogen alpha chain, complement C3, and complement C4) native serum peptides observed in our previous studies (Figure 4) [25-27].

The heavy peptide analogs were added to serum right after incubation in either water or guanidine HCl and the rate of peptide disappearance was evaluated at short (2 h) and prolonged (overnight) incubation times. The results show substantial degradation of all the tested peptides in serum diluted with water. The peptides have different susceptibility to proteolytic degradation, evidenced by the varying rates of degradation in water at the 2 h incubation time point. Stabilization of the peptides in serum requires denaturing conditions. The use of 6 M guanidine HCl stabilizes the peptides quite effectively even at the overnight incubation time. The stabilization of peptides in other body fluids still remains to be verified, but there is no reason to believe that proteolysis would not be inhibited to a similar degree; the 6 M guanidine HCl should provide effective starting conditions for the optimization of native peptide stabilization and recovery. We suggest that quantification of the native peptides in serum and other body fluids under standardized and validated analytical methods will clarify the controversies reported in the literature.

### Recovery of peptides of interest after denaturing ultrafiltration

Besides the total peptide yield we examined the recovery of three peptides of interest after denaturing ultrafiltration in guanidine HCl. The recovery of CO3 and CO4 peptides was 58% and 54%, FIBA peptide showed greater than 100% recovery most likely due to its high concentration in the serum that was used as the matrix. We thus repeated the experiment with a BSA solution instead of serum because albumin is the most abundant serum protein and its solution reasonably mimics the serum environment. This resulted in a recovery of 78%.

### Identification of native serum peptides using 2D LC-MS/MS and construction of an MS/MS spectral library

The goal of our study was to design effective LC-MRM MS quantification assays of native peptides. The design of assays for the FIBA, CO3, CO4 and other peptides is greatly simplified when information about the likely fragments of the peptides is available. Instead of considering and testing the many possible transitions for each peptide, we decided to build a MS/MS library of spectra to facilitate the design process. To this end, we processed serum samples using the optimized ultrafiltration procedure and fractionated the samples by a two dimensional high/low pH RP chromatography for MS/MS analysis using a QTOF mass spectrometer. The off line high pH step is orthogonal to the subsequent low pH nanoLC-MS/MS as described previously and leads to a significant increase in the number of peptide identifications [34]. The combined LC-MS/MS analysis of all the high pH fractions resulted in the identification of 186 protein groups (at ProteinPilot confidence 95%) on the basis of 12,762 distinct identified peptides. In order to further reduce the potential for errors in the spectral library, we kept only the highest confidence peptide identifications. After additional filtering of peptide identifications based on precursor mass-delta, reported post-translational modification, and score, the false discovery rate (FDR) of peptides included in the library was estimated as 1.23% at the spectra level, 3.61% at the ion level, and 4.30% at the peptide level.

Initially, we considered various modified peptides, including deamidated peptides, identified by ProteinPilot for the inclusion in the library; however, our analysis suggests that including these identifications significantly degrades the FDR of the resulting peptide identification set without significantly increasing the number of peptides or ions. The spectra were finally imported into a custom Skyline library (see Additional file 1) in order to create a generally available resource [24]. The final number of precursors imported in the library is 416, representing 349 unique peptide sequences from 83 proteins (parsimony assignment). Proteins with 10 or more unique native peptides include fibrinogen alpha chain (69), inter-alpha-trypsin inhibitor heavy chain H4 (26), complement C4-A (21), complement C3 (19), fibrinogen beta chain (15), apolipoprotein A-I (12), serum albumin (11), prothrombin (10) and zyxin (10).

**Figure 5:**
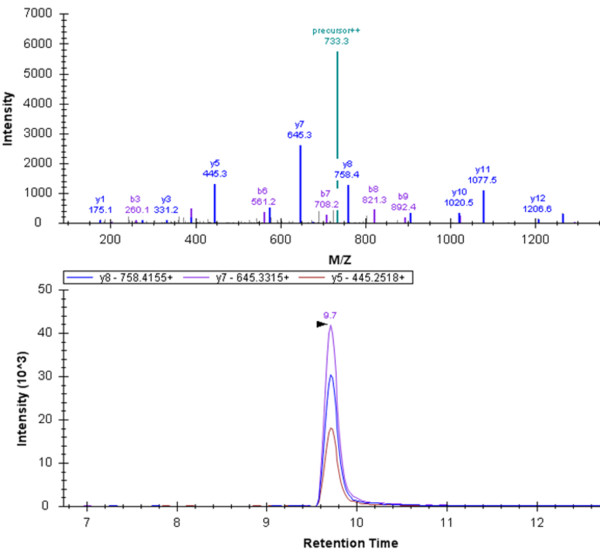
**Intensity of fragment ions of the doubly charged FIBA peptide in the MS/MS spectrum; the most intense fragments from the QTOF spectrum correspond to the three most intense 4000 QTRAP MRM transitions**.

The peptide fragmentation in the QTOF (QSTAR Elite, AB Sciex) and 4,000 QTRAP (AB Sciex) mass analyzers are expected to be similar and we find the fragmentation informative for the design of MRM transitions (Figure 5). The QTOF and QTRAP mass spectrometers are both manufactured by AB Sciex; differences in the peptide fragmentation in instruments from other manufactures may be more substantial. The lack of instrument-specific spectra is a significant concern, but long-term, we expect this to be resolved by sharing of MS/MS spectral information by research groups. The importance of PeptideAtlas for the design of MRM assays of tryptic peptides suggests that such as resource for native peptides should be considered [23]. The benefit of the library is perhaps best summarized by the following observation. To design MRM assays for the FIBA, CO3 and CO4 peptides, we have considered following peptide characteristics: precursor charges: 2, 3, 4; fragments charges: 1, 2; fragment types: y, b; first fragment - last fragment: 1 to last - 1; *m/z *from - to: 100-2000. A comprehensive testing of peptide fragments with these characteristics requires the consideration of 459 transitions for the CO3, CO4 and FIBA peptides. For tryptic peptides, attention can be focused on y-series fragments for Q3 filter masses, as these are most likely to provide sensitive transitions. This is because trypsin digestion results in peptides with the basic amino acids, arginine or lysine, at the C-terminus, leading to intense y-series ions formation under fragmentation. Furthermore, as tryptic peptides are short and usually carry limited number of basic amino acids, doubly charged precursor and singly charged fragments can be preferentially selected for MRM assays. Unfortunately, these heuristic rules cannot be extrapolated to native peptides. This substantially increases the number of potential transitions that must be considered and tested. In fact, for the CO3 peptide SSKITHRIHWESASLL in charge states +3 and +4, all of the selected transitions were doubly-charged b-ions. The MS/MS library allows us to select just five transitions for each precursor, resulting in the optimization of just 20 transitions. The spectral library turns the task of thoroughly optimizing 459 transitions for the quantification of three peptides into the much more reasonable task of checking 20 transitions. Furthermore, without the library, it is unclear when multiply charged or b-ion transitions should be considered, or y-ion transitions removed from consideration. In one case (SSKITHRIHWESASLL), a Lys residue near the N-terminus suggests b-ion transitions should be considered, but for another peptide (NGFKSHALQLNNRQI) with Lys residue near the N-terminus, the Arg residue near the C-terminus ensures that y-ion are abundant. For the peptide DSGEGDFLAEGGGVR, the y_6_-ion is much less abundant than y_7 _and y_5 _and should perhaps not be used.

### MRM analysis

To demonstrate the utility of the optimized workflow and the MS/MS library, we have designed LC-MRM MS assays for the quantification of the FIBA, CO3, and CO4 peptides in serum samples. These peptides were chosen based on our previous screens, which showed differences in the abundance of these peptides in the serum of hepatocellular cancer patients when compared to the serum of chronic liver disease patients and disease free controls [25,26].

**Figure 6:**
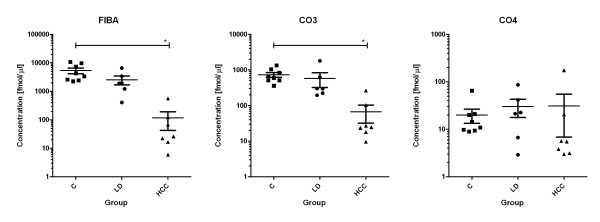
**Quantification of FIBA, CO3, and CO4 peptides in samples of healthy controls (C), chronic liver disease patients (LD), and hepatocellular cancer patients (HCC)**.

We first selected the five most intense transitions for each peptide based on the data in the MS/MS library. Collision energy was optimized for each transition and the declustering potential was optimized for each precursor. The three most intense transitions per precursor, after optimization, were selected for the FIBA and CO4 peptides; for the CO3 peptide, the two most intense optimized transitions from both triply and quadruply charged precursors were used (Table 1). We have further tested the linearity of the response for the selected transitions, assessed the potential cross-talk between light and heavy analogues of the peptides, evaluated potential background interference, and estimated the carry-over between chromatographic runs. The assays were linear over 3.4 orders of magnitude with limit of quantification 0.8 fmol/μl (FIBA, CO4) and 4 fmol/μl (CO3) (see). The cross talk was negligible and the carry-over was less than 1% due to the injection of a blank (50% trifluoroethanol and AcN) between samples. We did not observe a shift in normalized peak areas obtained from spiked heavy labeled peptides or native peptides in serum when compared to the expected values. This shows that the quantification is not affected by background interference. Based on these characteristics, we have adopted these optimized transitions for the absolute quantification of these peptides in serum samples. The optimized assays included denaturation of serum with 6 M guanidine HCl, ultrafiltration, desalting of the filtrate on the C18 SPE cartridges, and a triplex LC-MRM MS quantification as described in the methods. Total run time of the assay was 20 min with the average retention time of 9.78 min (FIBA), 9.61 min (CO3), and 9.32 min (CO4), respectively, for each of the three peptides (Figure 6).The absolute concentration of these peptides, as opposed to relative quantification in our previous studies, were determined in the samples of healthy donors (n = 8), patients with chronic liver disease (n = 6) and hepatocellular carcinoma (HCC) (n = 7). The heavy labeled peptide standards were spiked into the 8 M guanidine HCl solution that was added to each serum sample as the first step of the sample preparation. This minimizes the error introduced in absolute quantification that might result from differences in peptide degradation or recovery in the preparative steps.

**Table 1:** Optimized transitions selected for the quantification of FIBA, CO3, and CO4 peptides and their heavy analogues

Protein name	Peptide sequence	Form	Precursor *m/z *	Precursor charge	Product m/z	Product charge	Fragmention	DP	CE
**FIBA**	DSGEGDFLAEGGGVR	Light	733.33	2	758.42	1	y8	85	38.3
			
			733.33	2	645.33	1	y7	85	38.3
			
			733.33	2	445.25	1	y5	85	38.3
		
		Heavy	735.84	2	761.43	1	y8	85	38.3
			
			735.84	2	648.34	1	y7	85	38.3
			
			735.84	2	448.26	1	y5	85	38.3

**CO3**	SSKITHRIHWESASLL	Light	622.34	3	810.92	2	b14	80	27.9
			
			622.34	3	867.46	2	b15	80	26.4
		
		Heavy	624.68	3	810.92	2	b14	80	27.9
			
			624.68	3	870.97	2	b15	80	26.4
		
		Light	467.01	4	767.40	2	b13	70	21.7
			
			467.01	4	810.92	2	b14	70	17.2
		
		Heavy	468.76	4	767.40	2	b13	70	21.7
			
			468.76	4	810.92	2	b14	70	17.2

**CO4**	NGFKSHALQLNNRQI	Light	580.65	3	885.49	1	y7	85	29.6
			
			580.65	3	757.43	1	y6	85	31.1
			
			580.65	3	644.35	1	y5	85	31.1
		
		Heavy	582.99	3	892.51	1	y7	85	29.6
			
			582.99	3	764.45	1	y6	85	31.1
			
			582.99	3	644.35	1	y5	85	31.1

Kruskal-Wallis test with Dunn's post test confirmed significant differences in concentration of FIBA and CO3 peptides between cancer patients and the healthy and liver disease control groups

The FIBA and CO3 peptides appear significantly lower in the cancer patient samples compared to the chronic liver disease patients and healthy controls as observed in our previous studies. We did not confirm the increase in the CO4 peptide observed previously; the concentration of this peptide is substantially lower and the variation in measured abundance appears to dominate the magnitude of the change. It is possible that our previous MALDI-TOF based analysis was not sufficiently accurate, but we cannot, however, exclude other explanations like degradation of the peptide in storage. We want to emphasize that we are not making claims of biological importance or disease classification in this paper; we are presenting a workflow for native peptide extraction and the use of a MS/MS library of spectra for facilitating the design of quantitative MRM assays for native peptides.

## Conclusions

Accurate quantification studies of native peptides are expected to substantially increase our understanding of their function in biological processes. Mass spectrometry offers inviting possibilities for their quantification even in complex biological matrices like body fluids. The semi-quantitative screens based on relative quantification are increasingly being replaced by improved methods such as LC-MRM MS assays. In this paper, we present a workflow for the analysis of native peptides with emphasis on optimization of the enrichment protocol and characterization of the serum peptidome. We demonstrate the benefits of stabilizing serum samples with 6 M guanidine HCl as well as the added value of the workflow in the facilitated development of LC-MRM MS assay of peptides derived from fibrinogen alpha chain, complement C3 and complement C4. The publically available library of MS/MS spectra is expected to facilitate design of the MRM assays by a wider group of investigators. The triplex quantitative method has been evaluated on serum samples of 21 individuals in the context of the progression of liver disease to hepatocellular carcinoma.

## Methods

### Material

Native peptides derived from fibrinogen alpha chain, complement C3 and complement C4 were used in this study. The peptides and their isotopically labeled analogs were purchased from the Synthetic Peptide Application Lab at the University of Pittsburgh (FIBA, DSGEGDFLAEGGGVR) or Emory University Michrochemical Facility (CO3, SSKITHRIHWESASLL and CO4, NGFKSHALQLNNRQI). The DSGEGDFLAEGGGVR peptide analog carried one ^13^C isotope in each glycine residue, while the SSKITHRIHWESASLL and NGFKSHALQLNNRQI peptides carried six ^13^C and one ^15^N isotopes in a leucine at position 15 and 10, respectively. Guanidine hydrochloride, trifluoroacetic acid, acetonitrile, water, methanol, ^3^M Empore C18-SD cartridges, and ammonium bicarbonate were purchased from Sigma-Aldrich (St. Louis, MO). BCA assay, BSA standard, methyl methanethiosulfonate, and tris(2-carboxyethyl) phosphine hydrochloride (TCEP) were purchased from Pierce (Rockford, Il). Amicon Ultra-0.5 ml ultrafilters with 30 kDa MWCO membrane were from Millipore (Billerica, MA). Sequencing Grade Modified Trypsin was obtained from Promega (Madison, WI). Water Optima LC/MS, acetonitrile Optima LC/MS, and formic acid (FA) Optima LC/MS were purchased from Fisher Scientific (Fair Lawn, NJ). Guard column XTerra MS C18, 5 μm, 2.1 × 20 mm, analytical column XTerra MS 3.5 μm C18 2.1 × 100 mm, trap column nanoACQUITY UPLC 5 μm Symmetry C18, 180 μm × 20 mm, and nanoACQUITY UPLC column, 1.7 μm BEH C18, 75 μm × 150 mm were obtained from Waters (Milford, MA). Column ProSwift RP-1S, 4.6 × 50 mm column was purchased from Dionex (Sunnyvale, CA). PicoTip emitter tips were from New Objective (Woburn, MA). NuPAGE 10% Bis-Tris electrophoretic gels, running and sample buffers and XCell SureLock Mini-Cell were from Invitrogen (Carlsbad, CA).

### Blood collection, processing and serum handling

Samples were collected in agreement with established IRB protocols. Patients diagnosed with hepatocellular carcinoma were enrolled at the National Cancer Institute of Cairo University, Egypt, from 2000 to 2002 as described previously [27,35]. Individuals free of liver disease were recruited from the orthopedic department of Kasr El-Aini Faculty of Medicine at Cairo University and samples from the patients diagnosed with chronic liver disease were acquired at the Ain Shams University Specialized Hospital and the Tropical Medicine Research Institute, Cairo, Egypt as described [27,35]. Briefly, adults (age 18 and older) previously diagnosed with HCC and those without a prior history of cancer were eligible to participate in the study. The presence of HCC and chronic liver disease was confirmed by pathology, cytology, imaging and elevated levels of serum AFP. Blood samples were collected by a trained phlebotomist each day at around 10 a.m. and processed within a few hours according to a standardized protocol. Aliquots of sera were frozen immediately after collection and stored at -80°C until the time of analysis.

### Preparation of native peptides by denaturing ultrafiltration

Serum (100 μl) was diluted with water (300 μl), 8 M guanidine HCl (300 μl, final concentration 6 mol/l), or AcN (300 μl, final concentration 20%). The Amicon Ultra-0.5 ml ultrafilters with 30 kDa MWCO membrane were washed twice with 500 μl of 6 M guanidine HCl at 5.000 × g for 15 min. The remaining wash solution was removed by converting the filter upside down and spinning at 1.000 × g for 1 min. Samples were filtered at 8.000 × g and 30 min. Peptides were cleaned-up using C18 SPE cartridges; 3 M Empore C18-SD cartridges were washed with 0.5 ml of methanol and equilibrated with 0.5 ml of SPE solvent A (2% AcN, 0.1% trifluoroacetic acid (TFA)). After diluting 80 μl of the ultrafiltrates with up to 1 ml of the SPE solvent, samples were loaded onto the cartridges and trapped peptides were washed with 1 ml of SPE solvent A. Peptides were eluted with 0.4 ml of SPE solvent B (80% AcN, 0.1% TFA or 35% AcN, 0.1% TFA) directly into a new micro-tube. BCA assay and a UV-based HPLC assay were used to estimate the peptide yield in the ultrafiltrate. Each sample was prepared for this purpose in triplicate.

### BCA assay

SPE eluates were dried in a vacuum centrifuge and re-dissolved in 20 μl of 0.2% SDS solution. The BCA assay was used according to the instruction of manufacturer with minor modifications. A 5 μl aliquot of each sample was incubated with a 20 μl of a 50:1 ratio of the kit reagent A:B at 37°C for 1 h. Absorbance was measured against a BSA protein standard curve with each sample measured in triplicate. Samples outside the linear range were diluted accordingly. Absorbance at 562 nm was determined using a micro-volume UV-vis spectrophotometer ND-1000 (Thermo-Fisher, Wilmington DE).

### UV-based HPLC assay

This assay was used for relative comparison of the peptide yield. Fifty microliters of the ultrafiltrate were diluted 20× by RP solvent A (2% AcN, 0.05% TFA). For each replicate, 900 μl was loaded onto the column on HP 1100 HPLC system (Hewlett Packard, Waldbronn, Germany); we used a guard-column (XTerra MS C18, 5 μm, 2.1 × 20 mm) heated to 40°C at a flow rate of 1 ml/min with the peptides eluted by a step increase from 0% to 70% of RP solvent B (98% AcN, 0.05% TFA) in 0.01 min. The chromatogram was monitored at 214 nm and a blank chromatogram resulting from the injection of 10 μl of RP solvent A was subtracted. Area of all detected peaks was summed up as an estimate of peptide quantity. This allowed us to assess the quantity independently of any secondary reaction of the peptides required in the BCA assay.

### Recovery of peptides of interest after denaturing ultrafiltration

Light synthetic peptides FIBA (50 pmol), CO3 (5 pmol) and CO4 (2 pmol) dissolved in 45 μl of 8 M guanidine HCl were added to 15 μl of serum. The ultrafiltration was performed as described above. Ultrafiltrate (30 μl) was spiked with isotopically labeled analogs of FIBA (25 pmol), CO3 (2.5 pmol) and CO4 (1 pmol) and desalted on C18 SPE cartridges as stated above. The concentrated SPE eluate (approximately 50 μl) was mixed with 2.5 μl of a matrix solution (3.6 mg of CHCA/ml of 50% AcN, 0.1% TFA). From this mixture 0.7 μl was spotted in triplicate on an Opti-TOF MALDI sample plate and the MS spectra were recorded in relfectron mode on a 4800 MALDI-TOF/TOF mass spectrometer (Applied Biosystems/MDS Sciex, Foster City, CA). The intensity of peaks corresponding to *m/z *of non-labeled and labeled FIBA, CO3 and CO4 peptides were extracted and the ratio of light and heavy peptide analogs was calculated. The ratios were compared to ratios obtained from the direct mixture of light and heavy peptides prepared from the same starting solutions that did not undergo ultrafiltration. The recovery was calculated as the change in ratios determined in spiked ultrafiltrates of serum and directly in the peptide mixture.

### MALDI-TOF and HPLC-UV analysis of the denaturing conditions in a BSA/trypsin model

Eight microliters of a BSA solution (2.5 μg/μl) was added either to water, 20% AcN, or 6 M guanidine HCl as above. BSA was TCEP reduced, blocked by methyl methanethiosulfonate, and digested with 0.4 μg of trypsin in 100 mM ammonium bicarbonate buffer, pH 7.5, at 37°C for 40 h. Instead of the trypsin solution, water was added as a control. The reaction was stopped with the addition of 380 μl of the above RP solvent A. The results were evaluated using MALDI-TOF mass spectrometry and an HPLC analysis with UV detection. For MALDI analysis, 30 μl of the sample was desalted on 3 M Empore C18-SD cartridges using the same procedure as described above. The SPE eluates were concentrated in a vacuum centrifuge to approximately 20 μl. MALDI-TOF analysis was conducted in triplicate as described in the previous section. For the HPLC-UV detection, 200 μl was loaded onto a ProSwift RP-1S, 4.6 × 50 mm column. The thermostat was set up at 60°C and the flow rate was 1.8 ml/min. The gradient was from 0% to 75% B in 6 min. The chromatogram was monitored at 214 nm.

### Test of denaturing potential using three native peptides in serum

Serum (15 μl) was diluted either with 60 μl of water or 60 μl of 8 M guanidine HCl (final concentration 6 mol/l). Synthetic peptide standards were added to each serum sample as follows to achieve comparable detector response: 187.5 pmol of CO3 peptide and 37.5 pmol of CO4 peptide. The samples were incubated for two hours and a second batch of samples was incubated overnight. After incubation, isotopically labeled peptides were added to each sample in the following amount: 25 pmol of FIBA, 250 pmol of CO3 and 50 pmol of CO4 which gives initially an approximately 1:1 response (CO3 and CO4) and 6:1 response (FIBA) with respect to the light peptide. Negative control samples were prepared in exactly the same way but without the two hour incubation. Low molecular mass polypeptides including the added synthetic peptides were enriched by ultrafiltration as described above including the C18 SPE cartridge cleanup. MALDI-TOF analysis was conducted in triplicate as described above. The intensity of peaks corresponding to *m/z *of non-labeled and labeled FIBA, CO3 and CO4 peptides were extracted and quantified.

### 2D LC-MS/MS identification of native serum peptides

Serum (200 μl) diluted with 600 μl of 8 M guanidine HCl was kept on ice for 5 min. The remaining steps were as described above without spiking with the standard peptides. For each analysis, we combined two ultrafiltrates. First dimensional HPLC separation was an RP HPLC at pH 10.0. 200 mM NH_4_FA, pH 10, was added to the ultrafiltrates to achieve a final concentration of 20 mM NH_4_FA. The diluted sample was loaded onto an XTerra MS 3.5 μm C18 2.1 × 100 mm column. The thermostat was set at 40°C and the flow rate at 0.2 ml/min. The peptides were eluted by a 2% to 62% B in 60 min formed by basic RP buffer A (20 mM NH_4_FA, pH 10) and basic RP buffer B (90% AcN, 20 mM NH_4_FA, pH 10). The chromatogram was monitored at 214 nm. Starting at 5 min, 14 fractions were collected each 5 min. Peptides in each fraction were dried and re-dissolved in 75 μl of LC-MS solvent A (2% AcN, 0.1% FA) for an injection onto a nanoACQUITY system (Waters, Milford, MA). Peptides were pre-concentrated on a nanoACQUITY UPLC trapping column, 5 μm Symmetry C_18_, 180 μm × 20 mm at a flow rate 20 μl/min. The peptides were resolved on an analytical nanoACQUITY UPLC column, 1.7 μm BEH C_18_, 75 μm × 150 mm by a gradient formed by LC-MS solvents A and B (98% AcN, 0.1% FA) at the flow rate 300 nl/min. The linear gradient was from 2% to 40% B in 33 min with on-line detection using a Q-TOF tandem mass spectrometer (QSTAR Elite, Applied Biosystems/MDS Sciex, Foster City, CA) equipped with a nanoelectrospray ion source with a PicoTip emitter. The instrument was operated in the Information Dependent Acquisition (IDA) mode in which the MS spectrum was recorded for 1 s and the 4 strongest 2+, 3+, 4+ or 5+ ions were selected for MS/MS with Dynamic Exclusion feature enabled. The MS/MS spectra were recorded in the *m/z *range 150 - 2000 using Automatic Collision Energy and Automatic MS/MS Accumulation (max. accumulation was 0.7 s). Each sample was injected twice.

The spectral data was searched using ProteinPilot 3.0 (Applied Biosystems/MDS Sciex, Foster City, CA) using the Paragon algorithm, which uses a sequence-tag search strategy, facilitating non-specific peptide identification without inflating search times. The search was specified using the following parameters in the Paragon method: sample type - identification; cysteine alkylation - none; digestion -none; instrument - QSTAR Elite ESI, species - *Homo sapiens*; search effort - thorough. The data were searched against the UniProt protein database. The Proteomics System Performance Evaluation Pipeline incorporated directly in ProteinPilot 3.0 was used to conduct target/decoy searches facilitate initial FDR estimates.

### MS/MS spectral library

ProteinPilot results, including decoy hits, were output in XML format using the vendor supplied tool, group2xml. The resulting XML file was parsed using an in-house script to permit filtering of the peptide identifications for score and identified post-translational modifications; to remove decoy hit peptides; and to permit re-estimation of peptide, ion, and spectrum level FDR for the remaining identifications. This script was used to eliminate peptide identifications with identification confidence (ProteinPilot Probability) less than 0.95; precursor mass more than 0.2 Da from the theoretical monoisotopic mass or the mass corresponding to the isotope cluster peak associated with one, or two, ^13^C isotopes; and modifications other than oxidation and Pyro-glu on Glu and Gln. After filtering, the high-quality peptide identifications and their associated tandem mass-spectra, as provided in the XML formatted output of ProteinPilot, were reformatted as mzXML and pepXML, with corresponding faux spectral identifiers. Skyline's spectral library (University of Washington, Seattle, WA) build process reads the mzXML and pepXML file pair and constructs a spectral library suitable for Skyline's MRM design tools. For each peptide ion (distinct peptide sequence, charge and modification state), Skyline clusters repeat identifications and selects a representative spectrum that is, on average, most like the others in the cluster. Only the representative spectra are retained. After ProteinPilot analysis, 28,077 unfiltered peptide identifications were formatted as XML, and 1469 were retained by the above filters. After clustering, the spectral library held MS/MS spectra for 416 peptide ions, representing 349 native peptide sequences, for browsing and MRM assay design. The library, configured for Skyline, facilitates the design of LC-MRM MS assays for native peptides.

### LC-MRM MS assays

Chromatographic separations were performed on a nanoACQUITY system (Waters, Milford, MA). After loading of the samples from a 4°C cooled autosampler, peptides were pre-concentrated on a nanoACQUITY UPLC trapping column, 5 μm Symmetry C18, 180 μm × 20 mm at a flow rate 15 μl/min for one min. Fast gradient from 20% to 45% of LC-MS solvent B in 5 min at a flow rate 400 nl/min was used to resolve peptides on an analytical nanoACQUITY UPLC column, 1.7 μm BEH C18, 75 μm × 150 mm. Peptides were quantified on a 4000 QTRAP hybrid mass spectrometer (AB Sciex) equipped with a nanoelectrospray ion source with a PicoTip emitter. A spray voltage of 2400 V was used with a source temperature 150°C. The mass spectrometer was operated in MRM mode with first quadrupole (Q1) filtering with 0.7 and third quadrupole (Q3) filtering with 1.0 unit mass resolution. For all MRM analyses, 30 ms dwell time was used for each transition. MIDAS based enhanced product ion spectra were recorded with Q1 filter set to 1.0 unit resolution. Enhanced product ion spectra were recorded at a scan speed 4000 amu/s between 150 to 1200 *m/z*, with enabled dynamic fill time and Q0 trapping.

The five most intense transitions for each suitable precursor were selected based on data deposited in the MS/MS library using Skyline. LC-MRM MS chromatograms were obtained on samples of native peptide from serum. Detected peaks were subsequently validated by a MIDAS experiment. After we unambiguously confirmed the detectability of selected peptides in serum samples, synthetic peptides were used for confirmatory analyses and optimization of collision energy and declustering potential. Three most intense optimized transitions per precursor (FIBA and CO4) were used in the final method for peptide quantification. The confirmatory analyses of synthetic CO3 peptide revealed that the best intensity transition is produced from its quadruply charged ion. The final method for CO3 quantification therefore included two most intense optimized transitions from triply and quadruply charged precursors which represent four most intense transition overall. Carryover between injections as well as potential cross-talk between light and heavy equivalents of the peptides was subsequently examined. The final MRM method was examined for the linearity of response and the limit of quantification using relative dilution series experiments (see Additional file 2). Potential background interferences were assessed by comparing normalized peak areas of each precursor to be monitored. The values obtained from injections of heavy labeled peptides dissolved in LC-MS solvent A were compared with those originating from the spiked heavy labeled peptides as well as from the native peptides in the serum samples. The MRM data were processed and evaluated in Skyline.

Samples to be analyzed were prepared according to the protocol described above. Sera (20 μl) were mixed with 8 M guanidine HCl containing heavy labeled analogues for absolute quantification. To each microliter of serum, 500 fmol of the FIBA and CO3 peptides and 50 fmol of the CO4 peptide were added together with 625 fmol of peptide SSKITHRIHWESASLLR per μl of serum, added to decrease non-specific adsorption of the CO3 peptide. The amount of native peptides enriched from an equivalent of 2 μl of original sera was injected for the final analysis and each sample was measured in duplicate. The MRM spectra data files were imported into Skyline for evaluation. The quantity of the peptides was determined by comparison to the spiked internal standard. Data was exported to GraphPad Prism 5 (GraphPad Software, La Jolla, CA) for statistical evaluation.

## Abbreviations

AcN: Acetonitrile; BCA: Bicinchoninic acid assay; BSA: Bovine serum albumin; FA: Formic acid; FDR: False discovery rate; FIBA: Fibrinogen alpha chain; CO3: Complement C3; CO4: Complement C4; guanidine HCl or G-HCl: Guanidine hydrochloride; HCC: Hepatocellular carcinoma; MRM: Multiple reaction monitoring; TCEP: Tris(2-carboxyethyl) phosphine hydrochloride; TFA: Trifluoroacetic acid.

## Competing interests

The authors declare that they have no competing interests.

## Authors' contributions

JL planned the experiments, performed the majority of the experiments and contributed in drafting the manuscript; RL provided technical assistance and was responsible for MALDI-TOF analysis and data evaluation; NE was responsible for identification data evaluation and managing, created and characterized the MS/MS library and contributed in drafting the manuscript; RG conceived of the study, participated in its design and coordination, drafted the manuscript. JL, NE and RG critically reviewed the content of the manuscript before submission. All authors read and approved the final manuscript.

## Supplementary Material

Additional file 1 **MSMS library.blib**. Library of MS/MS spectra of serum native peptides that can be readily imported into the Skyline software for MRM transition design. Click here for file

Additional file 2 **Figure **1**.pdf. Dilution curves for three native peptides and the respective CV values (n = 3)**. Click here for file

## References

[B1] Li L Sweedler JV Peptides in the brain: mass spectrometry-based measurement approaches and challenges Annu Rev Anal Chem (Palo Alto Calif) 2008 1 451 483 10.1146/annurev.anchem.1.031207.113053 20636086

[B2] Mitchell JW Atkins N Jr Sweedler JV Gillette MU Direct cellular peptidomics of hypothalamic neurons Front Neuroendocrinol 2011 32 377 386 10.1016/j.yfrne.2011.02.005 21334363 PMC3165142

[B3] Thevis M Bredehoft M Kohler M Schanzer W Mass spectrometry-based analysis of IGF-1 and hGH Handb Exp Pharmacol 2010 195 201 207 20020366 10.1007/978-3-540-79088-4_9

[B4] Gomes I Dale CS Casten K Geigner MA Gozzo FC Ferro ES Heimann AS Devi LA Hemoglobin-derived peptides as novel type of bioactive signaling molecules AAPS J 2010 12 658 669 10.1208/s12248-010-9217-x 20811967 PMC2976993

[B5] Keay SK Szekely Z Conrads TP Veenstra TD Barchi JJ Jr Zhang CO Koch KR Michejda CJ An antiproliferative factor from interstitial cystitis patients is a frizzled 8 protein-related sialoglycopeptide Proc Natl Acad Sci USA 2004 101 11803 11808 10.1073/pnas.0404509101 15282374 PMC511055

[B6] McDonald WH Yates JR III Shotgun proteomics: integrating technologies to answer biological questions Curr Opin Mol Ther 2003 5 302 309 12870441

[B7] Zougman A Pilch B Podtelejnikov A Kiehntopf M Schnabel C Kurnar C Mann M Integrated analysis of the cerebrospinal fluid peptidome and proteome J Proteome Res 2008 7 386 399 10.1021/pr070501k 18052119

[B8] Ling XB Mellins ED Sylvester KG Cohen HJ Urine peptidomics for clinical biomarker discovery Adv Clin Chem 2010 51 181 213 20857622 10.1016/s0065-2423(10)51007-2

[B9] Kamphorst JJ van der Heijden R DeGroot J Lafeber FPJG Reijmers TH van Ei B Tjaden UR van der Greef J Hankemeier T Profiling of endogenous peptides in human synovial fluid by NanoLC-MS: method validation and peptide identification J Proteome Res 2007 6 4388 4396 10.1021/pr0704534 17929855

[B10] Amado F Lobo MJC Domingues P Duarte JA Vitorino R Salivary peptidomics Expert Rev Proteomics 2010 7 709 721 10.1586/epr.10.48 20973643

[B11] Shen Y Tolic N Liu T Zhao R Petritis BO Gritsenko MA Camp DG Moore RJ Purvine SO Esteva FJ Blood peptidome-degradome profile of breast cancer PLoS One 2010 5 e13133 10.1371/journal.pone.0013133 20976186 PMC2956627

[B12] Villanueva J Lawlor K Toledo-Crow R Tempst P Automated serum peptide profiling Nat Protoc 2006 1 880 891 10.1038/nprot.2006.128 17406321

[B13] Orvisky E Drake SK Martin BM Abdel-Hamid M Ressom HW Varghese RS An Y Saha D Hortin GL Loffredo CA Enrichment of low molecular weight fraction of serum for mass spectrometric analysis of peptides associated with hepatocellular carcinoma Proteomics 2006 6 2895 2902 10.1002/pmic.200500443 16586431

[B14] Chertov O Biragyn A Kwak LW Simpson JT Boronina T Hoang VM Prieto DA Conrads TP Veenstra TD Fisher RJ Organic solvent extraction of proteins and peptides from serum as an effective sample preparation for detection and identification of biomarkers by mass spectrometry Proteomics 2004 4 1195 1203 10.1002/pmic.200300677 15048999

[B15] Koomen JM Li D Xiao LC Liu TC Coombes KR Abbruzzese J Kobayashi R Direct tandem mass spectrometry reveals limitations in protein profiling experiments for plasma biomarker discovery J Proteome Res 2005 4 972 981 10.1021/pr050046x 15952745

[B16] Kawashima Y Fukutomi T Tomonaga T Takahashi H Nomura F Maeda T Kodera Y High-yield peptide-extraction method for the discovery of subnanomolar biomarkers from small serum samples J Proteome Res 2010 9 1694 1705 10.1021/pr9008018 20184378

[B17] Longo C Patanarut A George T Bishop B Zhou WD Fredolini C Ross MM Espina V Pellacani G Petricoin EF Core-Shell hydrogel particles harvest, concentrate and preserve labile low abundance biomarkers Plos One 2009 4 e4763 10.1371/journal.pone.0004763 19274087 PMC2651577

[B18] Davis MT Auger PL Patterson SD Cancer biomarker discovery via low molecular weight serum profiling-are we following circular paths? Physiol Rev 2010 56 244 247 10.1373/clinchem.2009.127951 19959624

[B19] Villanueva J Nazarian A Lawlor K Yi SS Robbins RJ Tempst P A sequence-specific exopeptidase activity test (SSEAT) for "functional" biomarker discovery Mol Cell Proteomics 2008 7 509 518 17986438 10.1074/mcp.M700397-MCP200

[B20] van den Broek I Sparidans RW van Winden AW Gast MC van Dulken EJ Schellens JH Beijnen JH The absolute quantification of eight inter-alpha-trypsin inhibitor heavy chain 4 (ITIH4)-derived peptides in serum from breast cancer patients Proteomics Clin Appl 2010 4 931 939 10.1002/prca.201000035 21137033

[B21] Kuhn E Wu J Karl J Liao H Zolg W Guild B Quantification of C-reactive protein in the serum of patients with rheumatoid arthritis using multiple reaction monitoring mass spectrometry and 13 C-labeled peptide standards Proteomics 2004 4 1175 1186 10.1002/pmic.200300670 15048997

[B22] Anderson NL Anderson NG Pearson TW Borchers CH Paulovich AG Patterson SD Gillette M Aebersold R Carr SA A human proteome detection and quantitation project Mol Cell Proteomics 2009 8 883 886 10.1074/mcp.R800015-MCP200 19131327 PMC2689772

[B23] Deutsch EW Eng JK Zhang H King NL Nesvizhskii AI Lin B Lee H Yi EC Ossola R Aebersold R Human Plasma PeptideAtlas Proteomics 2005 5 3497 3500 10.1002/pmic.200500160 16052627

[B24] MacLean B Tomazela DM Shulman N Chambers M Finney GL Frewen B Kern R Tabb DL Liebler DC MacCoss MJ Skyline: an open source document editor for creating and analyzing targeted proteomics experiments Bioinformatics 2010 26 966 968 10.1093/bioinformatics/btq054 20147306 PMC2844992

[B25] An Y Bekesova S Edwards N Goldman R Peptides in low molecular weight fraction of serum associated with hepatocellular carcinoma Dis Markers 2010 29 11 20 20826913 10.3233/DMA-2010-0721 PMC3552294

[B26] Goldman R Ressom HW Abdel-Hamid M Goldman L Wang A Varghese RS An Y Loffredo CA Drake SK Eissa SA Candidate markers for the detection of hepatocellular carcinoma in low-molecular weight fraction of serum Carcinogenesis 2007 28 2149 2153 10.1093/carcin/bgm177 17724376 PMC2204039

[B27] Ressom HW Varghese RS Goldman L An Y Loffredo CA Abdel-Hamid M Kyselova Z Mechref Y Novotny M Drake SK Analysis of MALDI-TOF mass spectrometry data for discovery of peptide and glycan biomarkers of hepatocellular carcinoma J Proteome Res 2008 7 603 610 10.1021/pr0705237 18189345 PMC2268625

[B28] van den Broek I Sparidans RW Schellens JH Beijnen JH Quantitative assay for six potential breast cancer biomarker peptides in human serum by liquid chromatography coupled to tandem mass spectrometry J Chromatogr B Analyt Technol Biomed Life Sci 2010 878 590 602 10.1016/j.jchromb.2010.01.011 20116351

[B29] Anderson NL Jackson A Smith D Hardie D Borchers C Pearson TW SISCAPA peptide enrichment on magnetic beads using an in-line bead trap device Mol Cell Proteomics 2009 8 995 1005 10.1074/mcp.M800446-MCP200 19196707 PMC2689780

[B30] West-Norager M Kelstrup CD Schou C Hogdall EV Hogdall CK Heegaard NHH Unravelling in vitro variables of major importance for the outcome of mass spectrometry-based serum proteomics J Chromatogr B-Analyt Technol Biomed Life Sci 2007 847 30 37 10.1016/j.jchromb.2006.09.048 17112795

[B31] Lowenthal MS Mehta AI Frogale K Bandle RW Araujo RP Hood BL Veenstra TD Conrads TP Goldsmith P Fishman D Analysis of albumin-associated peptides and proteins from ovarian cancer patients Clin Chem 2005 51 1933 1945 10.1373/clinchem.2005.052944 16099937

[B32] Hortin GL Shen RF Martin BM Remaley AT Diverse range of small peptides associated with high-density lipoprotein Biochem Biophys Res Commun 2006 340 909 915 10.1016/j.bbrc.2005.12.098 16386709 PMC1586118

[B33] Greening DW Simpson RJ A centrifugal ultrafiltration strategy for isolating the low-molecular weight (<= 25 K) component of human plasma proteome J Proteomics 2010 73 637 648 10.1016/j.jprot.2009.09.013 19782775

[B34] Gilar M Olivova P Daly AE Gebler JC Two-dimensional separation of peptides using RP-RP-HPLC system with different pH in first and second separation dimensions J Sep Sci 2005 28 1694 1703 10.1002/jssc.200500116 16224963

[B35] Goldman R Ressom HW Varghese RS Goldman L Bascug G Loffredo CA Abdel-Hamid M Gouda I Ezzat S Kyselova Z Detection of hepatocellular carcinoma using glycomic analysis Clin Cancer Res 2009 15 1808 1813 10.1158/1078-0432.CCR-07-5261 19223512 PMC2850198

